# Recent Advancements and Applications of Nanosensors in Oral Health: Revolutionizing Diagnosis and Treatment

**DOI:** 10.1055/s-0044-1792010

**Published:** 2024-12-30

**Authors:** Meghna Dewan, Deepti Shrivastava, Lata Goyal, Abdalwhab Zwiri, Areen Fareed Hussein, Mohammad Khursheed Alam, Kumar Chandan Srivastava, Sukumaran Anil

**Affiliations:** 1 Sudha Rastogi College of Dental Sciences and Research, Faridabad, Haryana, India; 2 Division of Periodontics, Department of Preventive Dentistry, College of Dentistry, Jouf University, Sakaka, Saudi Arabia; 3 Division of Periodontics, Department of Dentistry, All India Institute of Medical Sciences, Bathinda, India; 4 Department of Oral Surgery and Diagnostic Sciences, Faculty of Dentistry, Applied Sciences Private University, Amman, Jordan; 5 Division of Orthodontics, Department of Preventive Dentistry, College of Dentistry, Jouf University, Sakaka, Saudi Arabia; 6 Department of Dental Research Cell, Saveetha Institute of Medical and Technical Sciences, Saveetha Dental College and Hospitals, Chennai, Tamil Nadu, India; 7 Department of Public Health, Faculty of Allied Health Sciences, Daffodil International University, Dhaka, Bangladesh; 8 Department of Oral & Maxillofacial Surgery & Diagnostic Sciences, College of Dentistry, Jouf University, Sakaka, Saudi Arabia; 9 Department of Oral Medicine and Radiology, Saveetha Dental College and Hospitals, Saveetha Institute of Medical and Technical Sciences, Saveetha University, Chennai, Tamil Nadu, India; 10 Department of Dentistry, Oral Health Institute, Hamad Medical Corporation, Doha, Qatar, College of Dental Medicine, Qatar University, Doha, Qatar

**Keywords:** biosensor, nanotechnology, nanobiosensors, periodontal disease, restorative dentistry, nanomaterials, diagnostic nano-dentistry, biomarkers, implants

## Abstract

Advances in the field of nanomaterials are laying the foundation for the fabrication of nanosensors that are sensitive, selective, specific, cost-effective, biocompatible, and versatile. Being highly sensitive and selective, nanosensors are crucial in detecting small quantities of analytes and early diagnosis of diseases. These devices, operating on the nanoscale, detect signals, such as physical, chemical, optical, electrochemical, or biological, and then transduce them into a readable form. They show great promise for real-time, point-of-care, and home-based applications in health care. With the integration of wireless technology, these nanosensors, specifically biosensors, can potentially revolutionize therapeutic techniques. These advancements particularly impact the oral cavity, the primary entry point for various bodily substances. Nanosensors can transform oral and dental health practices, enabling timely disease diagnosis and precise drug delivery. This review examines the recent advancements in nanobiosensors, exploring their applications in various oral health conditions while discussing their benefits and potential limitations.

## Introduction

The revolution of nanotechnology has enabled nanosensors to monitor and detect minuscule concentrations of biomarkers for disease diagnosis. 1 2 Nanomedicine was first described by Robert A. Freitas Jr. in 1993. It involves using nano-sized particles for disease prevention, diagnosis, treatment, and health maintenance and improvement. 3 It leverages nanostructures and nanodevices to monitor and manipulate biological functions at the molecular level, incorporating nanoscale materials, devices, molecular medicine, and even molecular machines and nanorobots. 4 5 In the same vein, the emergence of nano-dentistry has promised a near-perfect suite of dental health solutions through nanomaterials, biotechnology, tissue engineering, and nanorobots. 6 Nanotechnology in dentistry is categorized into three disciplines: diagnostics, drug delivery, and tissue engineering. Recent advances in the field of nanotechnology have led to progress in the development of nanosensors with the aim of detecting any optical, physical, chemical, and mechanical changes. Nanosensors can detect different analytes by various sensing techniques including colorimetry, magnetic susceptibility, reflectance, impedance, fluorescence, and resonance. 7

The unique characteristics of nanoscale functional materials make them more sensitive and specific for biomedical diagnostic purposes. The presence or concentration of biological analytes, such as biomolecules, biological structures, or microbes, is determined by the use of instruments known as biosensors. Nanobiosensors are a subtype that merges biological and physical elements to interpret biological processes into electrical signals, leveraging expertise from chemistry, biology, and nanotechnology. In dentistry, nanosensors are used in diverse ways, including nanocomposites for dental caries detection, fluorescent probes, Au@Ag nanorods (Au- Gold; Ag-Silver), smart brackets in orthodontics, and capacitive sensors in implant dentistry. Nanosensors have shown promise in diagnosing life-threatening diseases such as cancer and common oral ailments like periodontal diseases, caries, and oral malodor. Their capability to detect analytes at an extremely low molecular level makes them instrumental in identifying diseases in their early stages or monitoring recurrence after disease. 8 9 Moreover, novel biosensors such as the miniaturized batteryless passive sensor system are used for wireless salivary pH monitoring. As the nanotechnology industry experiences exponential growth, the full extent of its potential remains uncharted. This review thus aims to compile the remarkable developments in nanosensors and their applications in various fields of dentistry.

## Nanosensors

**Fig. 1:**
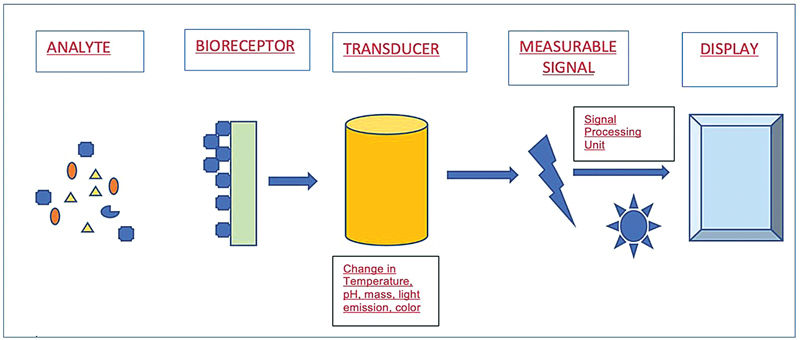
Working principle and design of biosensors.

Nanosensors are nanotechnology-based sensors that operate at the nanoscale, providing non-invasive bioanalytical capabilities for early detection of disease-specific markers. Their functionality is grounded in the principles of affinity and signal transduction. The nanosensors attach to the analyte based on its affinity which could be singleplex or multiplex. This attachment (ligand that has specificity) to an analyte is based on the various recognition elements, which could be proteins, enzymes or antibodies, forming a ligand–analyte complex. They use biophysical and biochemical properties of the biofluids to act. Further the signal is produced, after the analyte attaches to the sensor in the presence of electrolytes by physiochemical reaction ( Fig. 1 ). The intensity of this signal can be amplified depending on the types of nanoparticles (NPs) used and their sensitivity. 10 Various phenomena, such as optical or magnetic properties, serve as detectors to generate these signals. In the context of biosensors, a bioreceptor or acceptor is a biological element that attaches to a transducer or converter in various ways. Integrating biology and technology facilitates nanosensors' highly sensitive and specific detection capabilities.

### Characteristics of Biosensors

A biosensor is a device that leverages specific biochemical reactions mediated by isolated enzymes, immune system components, organelles, or tissues to detect chemical compounds. These detections are achieved by observing optical, thermal, or electrical signal changes. 7

**Fig. 2:**
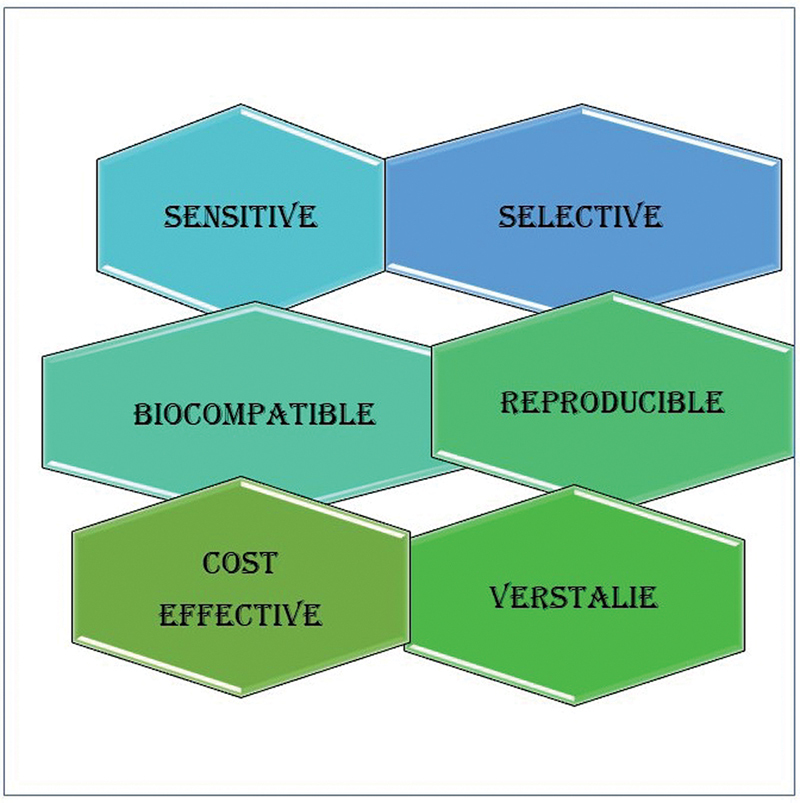
Characteristics of biosensors.

Apart from showing the properties of being lightweight, compact, sensitive, selective, easy-to-transmit signals, and stable, the sophisticated biosensors should be able to detect or monitor physiological changes in real for any clinical and diagnostic use ( Fig. 2 ). With advancing bioprinting technology, it will be possible to fabricate high-performance biosensors. A new wave of biosensor development is anticipated with the progressive strides in fabrication techniques. Such advancements would enhance the sensitivity and selectivity of these devices and enable their customization for specific applications or individual patient needs.

### Types of Biosensors

Biosensors have opened up opportunities for studying bodily phenomena and can be extensively used in translational research. From the perspective of this article, we shall examine numerous cutting-edge sensor technologies. 1 The various types of biosensors are classified based on the working principle and potential use ( Table 1 ). 8 9 10 11 12 13 14 15

**Table 1:** Working principles of the biosensors and its potential use

Types of biosensors	Principle
Electrochemical	Measures the current in biochemical reaction, which is produced after interaction of analyte and biological material due to difference in oxidation and reduction potential of test and reference electrode, e.g., early detection of cancer by morpho-differentiation of premalignant and malignant lesion by epidermal growth factor receptor (EGFR) expression
Optical	Based on the principle of fluorescence emission potential, e.g., different levels of fluorides in water can be measured by studying the variation in refractive index and shift of wavelength resulting in detecting dental fluorosis Fluorescent probes for detection of periodontal microorganisms
Electric	Difference in surface electrical potential resulting in alteration of conductance
Thermal	Heat generated from a biochemical reaction, e.g., multifunctional small dentures having microsensors that can detect strain, stress, and temperature
Magnetic	Changes in electromagnetic field Membrane with magnetic nanoparticle in periodontal pockets for the detection of inorganic ions
Chemical	Concentration of chemicals, e.g., clinical diagnostic purpose
Calorimetric	Changes in product color intensity resulting from biochemical reaction, e.g., serum α amylase point-of-care test for stress estimation
Physical	By measuring physical quantity Detecting mass, volume, pressure, and other physical properties

## Multidisciplinary Approaches for Developing Intraoral Biosensors

Numerous methods of health monitoring have been developed, taking into account the structural features and sensing environment of the organs they are intended to monitor. These tried-and-true methods also contribute to the development of tools that can be used on other body parts. 16 The mouth serves as a conduit between human and environmental systems. The mouth is a niche for a variety of distinctive biophysical and biochemical signals, making it a desirable area for the creation of monitoring devices.

Reduced invasiveness and resistance to an immune response are the key goals of material designs for implantable devices. These design principles and material advantages can also be applied to intraoral sensors that can prevent inflammation and can be implanted easily on tissues such as buccal mucosa and gingiva. Additionally, because saliva includes several macromolecules and metabolites that are present in blood and interstitial fluid, antifouling electrodes are also required for long-term monitoring.

The oral cavity has gained tremendous consideration as a target site for monitoring the disease activity. It serves as a crucial interface between the environment and human systems. In the oral cavity, saliva has been used extensively to screen the disease-specific biomarkers for developing a noninvasive and time- and cost-friendly diagnosis. These proven methods could be a rich toolbox for creating intraoral biosensors by utilizing the special characteristics of the oral cavity. 17

## Rationale of Using Intraoral Biosensors

Intraoral sensors are the medium for determining the oral health. The oral ecosystem is composed of various components that serve as the medium of communication between the environment and bionomics. Hence, the oral environment utilizes biophysical properties and biochemical properties to determine the innovation of intraoral sensors. 18

The tongue, teeth, palate, buccal mucosa, and other tissues are arranged in a highly specific way to support the biophysical function of the oral cavity. Every region serves a distinct and specific purpose. Numerous intraoral sensors are affixed inside the oral cavity without impairing the normal function of the mouth. There are distinctive biomarkers found in the oral cavity that are specific to it and are absent from the skin and internal organs. 19 Furthermore, inserting force detectors inside the brackets can produce accurate force profiles and speed up orthodontic procedures. 20

The intraoral biosensor may experience pressures from both the external environment (such as bacteria, food, and air) and within human systems (such as electrolytes, enzymes, and other metabolic processes). 21 22 Numerous systemic and environmental indicators are found in the oral cavity, which together with skin electronics and implanted devices provide a more complete picture of oral health. 22 23 24 25 26 27

## Biomedia Used in Oral Biosensors

The oral fluid such as saliva and gingival crevicular fluid (GCF) can be used as a biomedia for the oral biofluid-based biosensors. Since these fluids can be easily collected via noninvasive methods and are collected repeatedly, their usage in the detection of oral diseases has emerged as a potential diagnostic tool. 28

### Gingival Crevicular Fluid

The GCF is the inflammatory exudate or transudate produced from the gingival sulcus and the periodontal pocket. 29 It is composed of serum and various ions and enzymes, produced in health and disease. It is a biological media to identify and measure various biomarkers. 30 The different biomarkers in GCF indicate the absence or presence of disease. 29 Hence, the nanobiosensors are used to detect the biomarkers of health and disease. The technological obstacles involved in applying proteomic methods to characterize the molecular networks found in the GCF as well as their potential for identifying biomarkers are useful in therapeutic settings.

### Saliva

Saliva is crucial in maintaining the health of the oral tissues, interpersonal interactions, digestion, and prevention of oral infections. Saliva is considered a promising diagnostic tool. It has been used as a biomarker to determine any oral disease like periodontal disease and oral cancers. Saliva is composed of organic and inorganic components. The nanobiosensors are used to determine the change in pH of saliva and, any alteration in the composition of the saliva based on the ions, enzymes, and glucose can be interpreted and reflected as the presence of a disease. 31

## Nanosensors Used in Dentistry

### Nanosensors Used in Diagnosis of the Periodontal Disease

The early-stage identification of periodontal disease is made possible by the transformation of NPs into sensor-based materials. Periodontal disease is a chronic inflammation that affects a lot of patients of all ages. 32 33 34 It is one of the most common oral diseases that damages the periodontium and ultimately leads to tooth loss. 35 36 The various nanosensors used in the detection and management of periodontal disease have been explained in Table 2 .

**Table 2:** Applications of nanobiosensors used in the periodontal disease detection and management

Study	Type of nanosensors	Application
Totu et al 37	Sensor-based membranes. Sodium selective membranes with magnetic nano-inclusions using p-tertbutyl calix[4]arene as ionophore and polymerix (polyvinyl chloride)	This sensor can be used as a diagnostic tool for of the periodontal disease initiation and progression by measuring the ionic sodium level in the gingival crevicular fluid (GCF)
Fu et al 41	Hockey stick–shaped transducer	This transducer used photoacoustic imaging to detect periodontal pockets using a contrast agent (cuttlefish ink) and melanin nanoparticles, which enable broad photoacoustic absorption between 680 and 970 nanometers
Moore et al 43	Two optical fiber bundles integrated with both sides of a rectangular, linear array transducer	This transducer used photoacoustic imaging to detect periodontal pockets using a contrast agent (cuttlefish ink) that enabled broad photoacoustic absorption from 680 to 970 in the presence of melanin nanoparticles
Sheng et al 44	Nanobiosensor–based salivary biomarker detection	This is a noninvasive electrochemical biosensing method for detection of salivary cholesterol. Raised cholesterol levels may potentially increase the risk of developing periodontitis because they may accelerate alveolar bone resorption
Soni et al 42	Urea smartphone biosensor	A smartphone-based application that evaluates the levels of salivary urea levels, which in turn is helpful in detecting kidney diseases and periodontal disease
Xu 43	Titanium biosensors	This biosensor can diagnose the level of *Streptococcus gordonii* , which is a potential pathogen for periodontal disease. Its level can be measured pre- and postsurgeries and probably estimates the level of disease by estimating *S. gordonii*
He et al 38	Salivary biomarker–based biosensors	A lateral flow immunoassay based on upconverting nanoparticles was created to detect the periodontitis biomarkers in the GCF. MMP-8, IL-1β, and TNF-α demonstrated excellent sensitivity and specificity in GCF as well as synthetic saliva. It provides a quick diagnosis and reflects the correlation of these biomarkers with clinical parameters

#### New Sensor-Based Membranes

A new device is of pivotal role, wherein membranes with magnetic NPs and ionophoresis are capable of locally sensing the inorganic ions of interest in the periodontal pocket. Due to their potential to be remotely regulated based on a certain characteristic, namely their magnetic behavior, NPs included in the composition are predicted to help prevent implantable device failure. These new sensor-based membranes are used utilizing the ingredients of GCF like sodium, potassium, and calcium ions, which are an early indicator of periodontal disease. 37

#### Nanosensors Used in Oral Biofilm–Induced Diseases

**Fig. 3:**
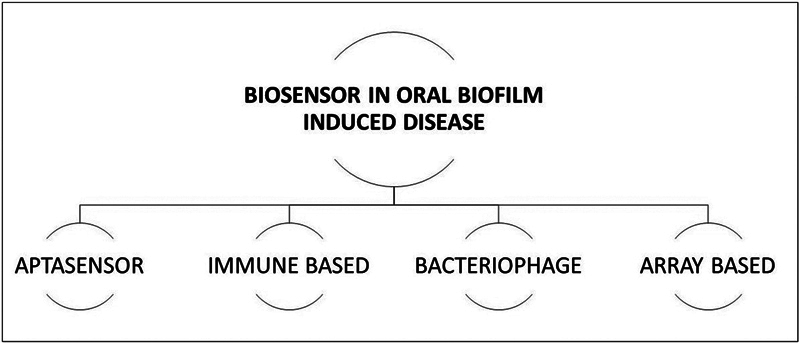
Biosensors in oral biofilm–induced diseases.

Nanosensors can be used in oral biofilm–induced diseases. 38 The use of NPs in the development of biosensors has given way to the creation of diagnostic tools for the detection of pathogenic bacteria. The various sensors used are Aptasensors and immune-, array-, and bacteriophage-based sensors ( Fig. 3 ). Bacterial detection is commonly used for plasmonic NPs such as gold and silver NPs with surface plasmon resonance (SPR) capabilities. Ultra-high-frequency optomechanical resonators are a new generation of nanosensors that can identify a single bacterium based on the mode of vibration of bacteria (piezoelectric sensor). The mechanism of these nanosensors is based on the frequency match of the disk and the bacteria. These nanosensors also give the details about the biological state of the bacteria. 39

#### Lateral Flow Immunoassay

Upconverting nanoparticles (G-UCNPs) are used as a luminescence probe in lateral flow immunoassay. Matrix metalloproteinase-8 (MMP-8), interleukin-1β (IL-1β), and tumor necrosis factor-α are all detected. 40 The lateral immunoassay strip was developed for chair side detection of periodontitis. The strip is disk-like in shape using green NPs as luminescence probe. This strip provides quick and accurate timely diagnosis of periodontal disease in the clinics. 40

#### Hockey Stick–Shaped Transducer

Photoacoustic imaging is a noninvasive imaging technique that combines ultrasound and laser-induced photoacoustic signals to create detailed images of the structures within the body. An innovative hockey stick–shaped transducer used in photoacoustic imaging detects periodontal pockets using a contrast agent (cuttlefish ink) and melanin NPs, which enable broad photoacoustic absorption between 680 and 970 nm. 41 42 These pockets can develop around the teeth where the gingiva has pulled away, often due to periodontitis. They are difficult to clean thoroughly and can become a breeding ground for bacteria, potentially leading to further tooth and bone damage. The hockey stick–shaped transducer is designed to fit comfortably inside the oral cavity and is used with photoacoustic imaging to detect these pockets. The detection process is enhanced by using a contrast agent such as cuttlefish ink or melanin NPs. When the light is absorbed, it generates a photoacoustic signal that the transducer can pick up, allowing for the detection and mapping of periodontal pockets. This technology represents a significant advancement in oral health care as it allows for the early detection of periodontal disease. This common condition can lead to tooth loss if left untreated. Moreover, it offers a noninvasive and potentially more comfortable option for patients than traditional probing methods. 43

#### Salivary Biomarker–Based Nanobiosensors

Salivary biomarker–based nanobiosensors are an exciting development in oral health diagnostics. These biosensors take advantage of the information in saliva to provide noninvasive and rapid detection of various oral and systemic health conditions. Cholesterol enzyme-immobilized platinum nanocluster is an example of this type of biosensor. This biosensor uses the enzyme to interact with specific cholesterol biomarkers in saliva. The enzyme and biomarker interaction causes a detectable change in the electrical signal, which can be measured and analyzed. Because of its nanoscale size and the high sensitivity of the platinum nanocluster, this biosensor can provide accurate measurements even when the biomarker concentrations are extremely low. 44 It is a noninvasive electrochemical biosensor. 45 Patients can provide a saliva sample easily and painlessly, and the fast-processing times mean results can be available quickly. This rapid, noninvasive testing can facilitate earlier diagnosis and treatment of diseases, potentially improving patient outcomes. 31

#### Mouthguard Uric Acid Sensors

Mouth guard uric acid sensors are used to determine the periodontal disease. The higher the salivary uric acid level, the less aggressive the periodontal disease. Mouthguard uric acid sensors represent a significant advancement in noninvasive medical diagnostics. These sensors are embedded in a device resembling a standard mouthguard that a patient can comfortably wear. The sensor within the mouthguard is designed to continuously monitor uric acid levels in the saliva. 46 It is hypothesized that uric acid may have antioxidant properties that can counteract the harmful effects of oxidative stress in the oral environment. The mouthguard uric acid sensor works by detecting changes in the concentration of uric acid in the saliva. This data can be collected and analyzed over time, providing a detailed picture of the patient's oral health. Regular monitoring of salivary uric acid levels can help identify individuals at risk of periodontal disease and allow for early intervention to prevent further disease progression. Such mouthguard sensors demonstrate the potential of wearable technology in health care. By allowing continuous, real-time monitoring of a patient's health markers, these sensors could revolutionize how we detect and manage diseases, moving toward more personalized and preventative health care approaches. 47

#### Smart Biosensors and Intelligent Devices

Smart and flexible designs that go beyond the conventionality of the biosensors enable real-time, rapid, and *in vivo* detection of salivary biomarkers. The characteristics of being portable, adaptable, varied, and affordable make the operation of the nanosensors much easier. The advancements are helpful in developing innovative salivary biomarker sensors. 48 The first segment is devoted to the creation of intelligent biosensors, which includes printing technologies, microfluidic chips, thin-film transistors, colorimetric strips, and self-designed biosensors. In particular, the highly integrated biodevices are the subject of the second segment. Examples include smartphone-based biosensors and portable mouthguard biosensors that can wirelessly communicate data and process it for analysis. In the near future, at-home testing or real-time monitoring for a variety of salivary biomarkers may be adopted as prospective diagnostic or screening techniques to enhance quality of life. Various studies have been conducted on the development of microfluidic and microelectromechanical systems for salivary diagnostics. This system carries out salivary diagnostics using tiny salivary samples and integrated detection techniques. Leucocyte chemotaxis is compromised by high blood sugar levels, which increase the chance of developing periodontal disease. 49 High salivary glucose levels cause plaque metabolism to produce too much lactic acid, which can increase the risk of dental caries. Platinum NP–coated organic electrochemical transistors can distinguish between the presence of lactate and glucose in saliva. Platinum nanoclusters, which are immobilized with cholesterol degrading enzyme, can be used for the detection of salivary cholesterol. Due to the potential of increasing alveolar bone resorption, high cholesterol can increase the risk of periodontitis. 44

### Nanosensors Used in Orthodontics

A microelectronic chip with numerous piezoresistive stress sensors is integrated with an orthodontic bracket as the basic concept of the smart bracket. Additionally, the orthodontist sets a screen reader next to each tooth during an examination, and this device receives wireless transmission of the measurement data. This provides the orthodontist with objective feedback. 50 51 Mechanical stress distributions in the bracket are produced by the combined force and torque components of the six degrees of freedom archwire. Utilizing the silicon's piezoresistive phenomena, microsensors inside the bracket detect stress in a sensor chip's plane. 52

The first telemetric stress-sensor device, often called as a telemetric smart bracket (TSB), was created specifically for orthodontic purposes. Thirty-two piezo-FET (field effect transistor) components make up the complementary metal oxide semiconductor (CMOS) chip. A 5-bit multiplexer (MUX) is used to sequentially connect the 32 sensors to the readout unit. Hafner et al used copper electroplating on glass substrates to construct a planar spiral-shaped micro-coil for inductive coupling. Flip-chip bonding is used to link the chip and coil assembly, which has an area of 2 × 2.7 mm ^2^ . The reader units, attached to a graphical user interface, demodulates, digitizes, and decodes the sensor data that have been extracted telemetrically. Resolutions better than 60 mN and 0.14 N mm were successfully used to measure the values of force and moment. 53

### Nanosensors Used in Restorative Dentistry and Endodontics

#### Fluorescent Ratiometric pH-Sensitive Microsensors

Using fluorescent ratiometric pH-sensitive microsensors to map the four-dimensional (4D) pH has a pivotal role in the detection of *Streptococcus mutans* biofilms. The rationale for using such technologies is to examine potential techniques to stop the acidification of oral biofilms and eventual demineralization of the enamel; this would reduce dental caries and enhance the standard of living for people who are affected. A potential use for the real-time monitoring of sugar metabolism by nanosensors is the discovery of new therapeutic approaches to enhance dental health. 54 55

#### Biotransferrable Graphene Wireless Nanosensor

Pathogenic bacteria can now be monitored with the advent of graphene nanosensors, wherein the bacteria on tooth enamel can be detected well before the initiation of the disease. This nanosensor was named a tooth tattoo. 56 The device has several important and distinguishing qualities as a sensing system, including extraordinarily high sensitivity provided by the graphene network, biotransferability provided by the water-soluble silk fibroin platform, broadly selective biorecognition made possible by the antimicrobial peptides (AMPs) and the capacity to create a wireless, battery-free remote sensing device. As a result, the right preventive actions can be designed to prevent caries. Even at the level of a single cell, precision and bioselectivity exist, making it useful for detecting dental caries. 56

#### Wearable Fluorescent Mouthguard Sensors

Hidden dental caries is closely related to the local generation of volatile sulfur compounds from anaerobic bacteria's degradation of sulfur-containing proteins. 57 58 The tentative diagnosis of latent dental caries was validated by closely observing the local emission of volatile sulfur compounds. Finding the hidden dental cavities was made feasible by the mouthguard sensor, which aided in the early identification, prevention, and treatment of dental caries. 59 Various NPs can be incorporated to make novel nanocomposites for the therapeutic purpose of dental caries. 60 Many NPs have been used in nanosensors for the diagnostic purpose.

#### Theranostic Dental Patch

This nanosensor is a double-layered structure used as a dental patch. This patch is used for controlled drug delivery like fluorides and is an efficient dental caries monitoring and treatment. 59 The dental patch offered a revolutionary approach to clinical and familial caries prevention since it could identify and treat the unseen lesion early on rather than waiting for the development of a cavity.

#### Au@Ag Nanorods

The wearable mouthguard Au@Ag nanorods-polydimethylsiloxane (NRs-PDMS), which is comprised of Au@Ag nanorods and polydimethylsiloxane, can be used to detect dental lesions by inferring the change in color. The Au@Ag NRs-PDMS composite exhibits distinct color change in response to hydrogen sulfide (H _2_ S) gas generated by bacterial breakdown at the lesion sites. 61 Additionally, it is demonstrated that the Au@Ag NRs-PDMS mouthguard possesses the necessary mechanical properties, excellent chemical stability, superb biocompatibility, and the capability to precisely localize the lesion areas within the human oral cavity. Thus, the mouthguard can be widely utilized to support people in self-monitoring their dental health in daily life.

### Nanosensors Used in Implant Dentistry

#### Capacitive Sensor

The bone growth around the dental implant can be observed using capacitive nanosensor and it has advantages over X-ray imaging. It is inserted in the bone to monitor the growth of new bone. The novel capacitive sensor was developed to prevent radiation exposure. The sensor created was a zero-power module utilizing less energy, dependable operation, and quick processing, fits easily within dental implants, and does not need any active components. The capacitive sensor transmits data to a reader device using a wireless inductive link. The findings of the experiments are supported by results from the COMSOL (computer and physics) sensor simulation. The built-in sensor was tested on the femur (the thigh bone) and mandible (the lower jaw). The sensor capacitance varies from 20 nF to 1.57 F depending on how the sensor was made and how much bone is around it. The sensor capacitance varies by more than seven times from the start of the dental implant through full healing and bone development, according to fabrication data. The change in the wide range of sensor capacitance made it feasible to characterize bone growth more accurately. 62

#### Temperature Sensor

These sensors can be implanted in the mouth and can instantly transmit warning signs before the implant fails. Implantable sensors with real-time measurement capabilities are useful tools for maintaining health and diagnosing illnesses because they provide routine or ongoing biometric monitoring. The polymer film demonstrated linear reversibility when put under stress by cyclic variations in temperature and humidity. The sensor maintains its stability in various stress situations brought on by changes in temperature, acidity, bending, and stirring. 63

#### Inbuilt Salivary Nanosensors in Dental Implants

Dental implants can have inbuilt nanosensors to detect acute myocardial infarction. The basic idea behind the disclosed gadget is saliva analysis for near heart attack detection. This saliva sensor is designed to seek for certain cardiac signs that are as little as possible. The main objective is to integrate this saliva sensor into a dental implant that will stay in a patient's mouth permanently and who is at high risk of a heart attack. The saliva sensor can perform a variety of reactions to assess for the presence of the most obvious cardiac indicators. Future examinations will offer a wider range of responses and much higher test standards. 64

### Nanosensors Used in Oral Medicine and Oral Surgery

#### Photoacoustic Imaging

In a metastatic oral squamous cell carcinoma mouse model, lymph node micrometastases are identified using ultrasound-guided spectroscopic photoacoustic imaging. Through the use of anti-epidermal growth factor receptor (anti-EGFR) antibody associated molecularly activated plasmonic nanosensors (MAPS), the study showed how MAPS changed their absorption spectra to the near-infrared region. Furthermore, within 30 minutes of MAPS injection, significant ultrasound-guided spectroscopic photoacoustic signals began to appear in micrometastases as small as 50 mm. These results provide an alternative method for inspecting sentinel lymph node biopsy samples obtained following mouth cancer resection. 65

#### Optical Biosensors

There are three types of optical biosensors used in the early detection of various biomarkers in the oral cavity using saliva as a media. The optical biosensors are fluorescent biosensors, microfluid biosensor, and fluorescent microsensors. These are the noninvasive optical technologies used in detecting a disease using biofluids. This innovative approach basically helps in the early detection of oral cancer. 66 Numerous factors must be considered when developing optical nanobiosensors, including but not limited to minimal background fluorescence, low photodamage to the virus oligonucleotide hybridization with the probe, high photostability, and low phototoxicity of the probes. In addition, concerns regarding toxicity, reproducibility, and throughput of the biosensors must be addressed. 67

#### Oral Fluid Nanosensor Test

The oral fluid nanosensor test (OFNASET) utilizes advanced nanotechnology to detect specific biomolecules present in saliva, offering insights into both oral and systemic health. This noninvasive diagnostic method offers high sensitivity capable of identifying biomarkers at incredibly low concentrations. Due to its rapid response time, the test provides near-instantaneous results, making it ideal for point-of-care applications. 68 For the purpose of finding salivary biomarkers for oral cancer, the OFNASET is used. The University of California, Los Angeles (UCLA) created this test, which may measure as many as eight biomarkers in less than 15 minutes. This helps determine salivary protein biomarkers and genetic markers with minimal discomfort to the patient. 68

#### Nano Biochip Sensor

Due to their nanoscale features, nano biochip sensors can achieve much higher sensitivity than conventional diagnostic methods. They hold promise for applications ranging from medical diagnostics to environmental monitoring. The integration of these sensors into portable devices can revolutionize point-of-care testing, allowing for immediate results in diverse settings. Exfoliative cytology, based on a nano biochip sensor platform, is used to detect oral cancer with 97 to 100% sensitivity and 86% specificity. Exfoliative cytology provides a quick, noninvasive way to sample epithelial cells from premalignant lesions, which are then analyzed microscopically or molecularly for indications of malignant transformation. 69

#### Deoxyribonucleic Acid Biosensor

Deoxyribonucleic acid (DNA) carries the genetic information and is the building block of heredity. DNA sensors were developed because they are less time-consuming as they overcome the need of enzyme-linked immunosorbent assay (ELISA) and polymerase chain reaction (PCR) for DNA sequencing used to detect the disease. Hence, there is significant reduction in time, effort, and cost. The DNA biosensors are used for early detection of oral cancer from saliva. The various advantages of DNA biosensors are high specificity, sensitivity, ultra-high discrimination capability, detection of low concentration of biomarkers, detection of multiple biomarkers at the same time. 66

#### Ribonucleic Acid Biosensor

Ribonucleic acid (RNA) biosensors use magnetic beads for sensitivity and distinguishability. It works on the principle of hybridizing the RNA sequence in the solid substrate, which is then immobilized with the immobilized probe, which has the complementary sequence we want to detect. 70 The RNA biosensors detect the cancer at a stage when there are no symptoms of the disease. The limit of detection for early-stage cancer is found to be 0.22 aM . Magnetically controllable biosensors use RNA-based sensors and have high sensitivity. 66

#### Protein Biosensors

Protein biosensors are used to detect protein cancer biomarkers. The surface of the electrodes is covered with antibodies or aptamers, and thus is an electrochemical nanosensor. 71 These biosensors are another noninvasive method of oral cancer detection. Multiplexed electrochemical sensors are protein-based nanosensors to detect biomarkers.

#### Nanoparticle-Based Biosensors

Salivary biomarkers using NPs like graphene and carbon nanotubes to detect the wide ecosystem of saliva are NP-based biosensors (nano platform). The chemical and functional alteration creates a hybrid product to recognize a range of substances in saliva. The salivary plethora is composed of hunger hormones, cytokines like IL-1, IL-6, IL-8, uric acid, ketamine, cardiac troponin I, cortisol, and biomarkers for DNA oxidative damage. 72 Extensive studies have also been done on the potential salivary biomarkers of oral cancer, including members of the cytokeratin family like cytokeratin-19. A potential technique to detect oral squamous cell carcinoma is done by using silicon nanowire transistors. 71 A lateral flow immunoassay was created to detect the periodontitis biomarkers in the GCF. MMP-8, IL-1β, and tumor necrosis factor-α (TNF-α) demonstrated excellent sensitivity and specificity in GCF as well as synthetic saliva. It provides a quick diagnosis and demonstrates significant connections with clinical procedures. 73

## Other Uses

### Dental Retainer Glucose Sensor

This a mouthguard type of biosensor also known as cavity sensor fabricated on a plastic substrate mounted in the oral cavity. This is a noninvasive method of saliva analysis. These advanced biosensors will be of great utility to diabetic patients. 74

### Pacifier Platform for Glucose Sensing

There has been a concept of oral point-of-care testing. To validate this concept, a noninvasive oral mouthguard was used to investigate the glucose levels in saliva. The pacifier was made of nontoxic silicone (saliva collector), which is then connected to the biosensor to produce an electrochemical activity. Thus, this was based on amperometric circuit. This nanosensor detects glucose based on the glucose oxidase transducer. 75

### Intraoral Electronic Biosensor for Sodium Intake Monitoring

This biosensor was developed by Lee et al to measure the sodium ion levels in the oral cavity. This biosensor was attached to an orthodontic bracket α-amylase imprinted intraoral biosensor. In this nanosensor, an amperometric transducer was used to quantify the levels of α-amylase. 76

### Electrochemical Nanobiosensors

These nanobiosensors are used to detect coronavirus disease (COVID) due to their improved electrical properties, chemical stability, sensitivity, and specificity. Gold NPs coupled with iron oxide NPs are also used in electrochemical nanobiosensors. Additionally, graphene functionalized with p-sulfocalix arene is used to detect ultrasensitive RNA from severe acute respiratory syndrome coronavirus 2 (SARS-CoV-2) with less time and expense. 77 Instead of utilizing antibodies or immunoglobulins to detect the COVID-19 virus, newer biomarkers could be used to build a new sensor for COVID-19 detection. This could evolve to be a revolutionary innovation.

## Advantages

The size of nanosensors and their special properties have led to this emerging era of intraoral nanosensors. The high specificity and sensitivity to detect the disease at an early monitoring is of obvious advantage. 78 These special properties in nanosensors over the conventional diagnostic and therapeutic methods have helped overcome some of the limitations. The conventional methods like ELISA and PCR, which detect the microbiota or the presence of a disease, take at least 6 to 24 hours to report over the nanosensors, which proved to be efficacious in early detection of disease and further accordingly plan a treatment to prevent its progression. These tests require adequate manpower and training along with various expensive reagents to perform a test. Thus, the nanosensors overcome all such needs. Another advantage of nanosensors is that they can be attached to the living cells, molecules, viruses, and proteins to monitor the early stage of a disease over the conventional methods, hence exhibiting various biophysical and biochemical reactions leading to a diagnosis. Another advantage of nanosensors is the low energy consumption data transmission technology, which makes oral point-of-care testing highly beneficial.

## Challenges

The oral cavity performs various activities in association with the microbes, ions, and enzymes. Thus, it performs various functions like eating, swallowing, and breathing, which could hinder the activity of the nanosensors. The other challenge is the expense and limited availability and utility of nanosensors. As there are various nanosensors like enzyme based on detection by physical method, other challenges were also observed. The chemical- or enzyme-based results could be hampered or not correctly monitored due to the fragile enzyme or very low concentration of bacteria. The other challenge was based on the life of the nanosensor and the service cost of the nanosensor. As the nanosensors depend on Bluetooth and radiofrequency for data transmission and also on battery or wireless transmission for power supply, one has to be very active for the needs for real-time monitoring. Finally, the detection limit varies in each nanosensor and they may not respond if the accurate load of bacteria is not present.

## Future Directions

The innovation and development of the nanosensors have been life changing and will evolve more with time. The advancement in technology is based on the size, low energy consumption, and personal health care potential with the oral point-of-care testing, thus instant health monitoring. Artificial intelligence, deep learning, and machine learning work together to produce various advanced ultrasensitive nanosensors. In the coming years, development and research will lead to more user-friendly nanosensors. The high specificity, sensitivity, and eco-friendly approach will make more desirable nanosensors. The development of dynamic nanosensors with multivariate properties to detect multiple pathogens or disease in the form of implantable chip or nanosensors at the same time could be life changing and revolutionary.

## Conclusion

This study has illustrated the advances in nanodentistry. It is expected that nanotechnology will continue to deepen and develop and find further applications in the scientific study of health. This prediction is supported by the potential benefits and cost-effectiveness, the benefits to the public, the workplace and the social environment, and the risks associated with nanodental research. The nanosensors work with different methods and, therefore, play a decisive role in recognizing pathogens and toxins and, thus, in rapid detection and early prevention. More research in this area could lead to promising and affordable diagnostic devices. The introduction of devices for use at the bedside and at the point of care is necessary. This is a long road, as design, function, and applications will evolve over time. More optimized devices will be the promising future. Continuous committed research on nanosensors should be conducted to remove hindrances, if any, followed by safety protocols and marketing for worldwide acceptance.
